# Enhancing primary care through integrated care pathways: a convergence of theory and practice

**DOI:** 10.3389/frhs.2024.1432901

**Published:** 2024-12-12

**Authors:** Waseem Jerjes, Daniel Harding

**Affiliations:** ^1^ Research and Development Unit, Hammersmith and Fulham Primary Care Network, London, United Kingdom; ^2^ Faculty of Medicine, Imperial College London, London, United Kingdom

**Keywords:** integrated care, primary healthcare, general practice, secondary care, National Health Service

## Introduction

Integrated care pathways (ICPs) represent an innovative development in healthcare provision, particularly in the primary care setting (1). A care pathway is a complex multidisciplinary plan of care that stipulates the necessary steps for managing patients with particular clinical conditions, thus standardising care across settings (2). ICPs bear special importance in the primary setting due to the variability in the needs of different patients, which requires their care to be both individualised and coordinated.

ICPs allow the integration of standardised processes, which might improve the quality of care and outcome, enhancing efficiency (1). Key challenges in ICP implementation include professional resistance and information and communications technology (ICT) system interoperability. This viewpoint provides deeper insight into ICPs by discussing how innovations can successfully overcome these barriers and present new, unexplored strategies to further enhance the effectiveness of ICPs in primary care.

### Theoretical framework and practical applications of ICPs

ICPs form the foundation of care that is systematic, evidence-based, and multidisciplinary. The ideal goal of an ICP is to manage healthcare provision to standardised levels while ensuring that care remains personalised to meet the peculiar needs of each patient. This model assists in fostering a systematic approach to health conditions; this becomes quite easy, especially in primary health settings where the needs of patients can be highly variable. By outlining specific steps in the care process, ICPs provide healthcare professionals with a consistent framework that guides the entire patient journey—from the initial diagnosis to treatment and long-term follow-up—ultimately reducing variations in care and enhancing patient safety (3, 4) (Table 1).

**Table 1:** Integrated care pathway for mild cognitive impairments in primary care.

Stage of care	Action	Involved specialties	Expected outcomes	Challenges	Potential solutions
Initial assessment	Patient screening using cognitive assessment tools	General practitioner (GP), nurse practitioner	Early detection of cognitive impairments	Lack of screening tools in primary care settings	Provide GPs with access to validated screening tools
Diagnosis confirmation	Referral to a neurologist for detailed evaluation	GP, neurologist	Accurate diagnosis confirmed	Delays in referral processes	Streamline referral processes through integrated health systems
Care planning	Development of a personalised care plan	Multidisciplinary team including a GP, neurologist, psychiatrist, social worker	Tailored care approach based on individual needs	Resistance to multidisciplinary approaches	Training sessions to highlight the benefits of integrated care
Implementation	Execution of the care plan, including medical and lifestyle interventions	GP, nurse practitioner, dietitian, physiotherapist	Improved patient health and wellbeing	Insufficient staff or resources	Leverage telehealth to extend care capacity
Monitoring and adjustment	Regular follow-ups to assess progress and adjust care plan as necessary	GP, nurse practitioner, psychiatrist	Continuous monitoring of patient condition	Lack of follow-up due to patient non-compliance	Implement reminder systems and patient education
Outcome evaluation	Evaluate the effectiveness of the care pathway	GP, entire care team	Documented improvement or stabilisation of cognitive status	Inconsistent documentation practices	Standardise documentation using EHRs

This structured approach has a particular application in managing chronic conditions, which by nature require frequent monitoring and change in treatment. ICPs guarantee uniformity in guidelines, as all health professionals involved in the care of a patient work on the same evidence-based protocol; this leads to a reduction in the chances of errors or omissions in the care provided. It involves combined efforts; for example, in diabetes, general physicians, endocrinologists, dieticians, and pharmacists are involved. This will lead to good coordination and enhanced control of the blood glucose levels as it helps the patient in long-term outcomes (5). The structured communication by ICPs also allows early identification of potential complications, thereby enabling timely intervention and preventing hospitalisation (6).

ICPs not only optimise clinical outcomes but also enhance efficiencies in health systems by considering resource utilisation. When applied to specific clinical pathways, such as elective surgeries or chronic disease management, ICPs manage to support health providers in arriving at informed decisions on the most appropriate use of resources such as staff time, medication, and utilisation of beds within hospitals. Oosterholt et al. (1) showed how ICP pathways can manage care effectively by reducing lengths of stay in hospitals and making sure every intervention is necessary and not delayed. This is very important in surgical care since exact coordination between the pre-operational, intra-operational, and post-operational care groups can prevent unnecessary delay and optimise recovery times. This represents how ICPs not only help raise care quality but also contribute to the economy, and thus, health administrators concerned with efficiency will consider ICPs a very welcome solution.

Despite these advantages, there are still challenges that ICPs face in their integration into practice. One of the major criticisms by health professionals is the perceived rigidity of ICPs, which may inhibit their judgment in practice (7). Resistance to ICPs partly stems from concerns that standardised care protocols cannot always be flexible enough to meet the needs related to the preference of individual patients or complex medical conditions. While ICPs are meant to be flexible, striking a balance between standardisation and customisation remains a thorny issue. ICPs can only hope to deliver their promise in primary care brass section if healthcare providers are actively involved throughout their development and refinement, including a balanced approach between evidence-based care and individualised treatment (8).

### Professional resistance to ICPs

Professional resistance is one of the most important impediments to the successful implementation of ICPs in a primary care setting. ICPs make health providers, especially physicians, feel that this damages their clinical autonomy. The rigid protocols introduced by these pathways in current clinical practice limited professionals' ability to make individualised decisions according to the needs of the patients (9). This tendency for a perceived loss of autonomy can lead to frustration, which is, in turn, precipitated when clinicians feel coerced into a “one-size-fits-all” model that fails to take account of the subtleties of clinical reality as it is experienced in everyday practice. Many clinicians indeed believe that while ICPs standardise care, they reduce the latitude required to manage patients with complex, multivariate diseases—a multitude of whose examples are seen in primary care (10, 11). This perceived rigidness results in professional dissatisfaction because health professionals feel that their expertise and judgment are being belittled for process standardisation.

Another layer of resistance in this regard is related to the fact that some administrative workloads, commonly imposed by ICPs, may be burdensome. Added documentation, compliance requirements, and communications among multidisciplinary teams tend to increase non-clinical tasks that many healthcare providers feel detract from patient care. According to Rathod et al. (4), clinicians involved in psychosis care also found the ICP protocols too rigid, thus dampening their ability to make fine-tuned, patient-specific decisions, while administrative tasks associated with the ICP added to their frustration. In the same line of thinking, Lalani et al. (2) also commented on how many clinicians consider ICPs to further bureaucratise practice, which will only result in more administrative burdens without necessarily impacting the delivery of quality patient care. This feeling is certainly common within busy primary care settings where providers are already at their limits and may more likely view an ICP as added layers of complexity rather than a practice tool to facilitate ease in delivering care (12).

Overcoming professional resistance to pathways requires a collaborative strategy, which involves healthcare providers in the design and operation of ICPs. Early involvement in an ICP tends to promote views among clinicians that these pathways are supportive tools that enhance rather than limit clinical practice. A number of studies have reported that involving clinicians in developing care pathways can increase their sense of ownership and decrease resistance to adoption (13, 14). In addition, efforts to highlight how ICPs would allow practitioners to reduce cognitive load, make decisions more quickly and effectively, and manage patients more efficiently can ease some concerns about the perceived administrative burdens (15). Assuring providers that ICPs serve as dynamic frameworks to support individualised care, rather than inflexible protocols, may increase their willingness to adopt these pathways for the benefit of their practice and for patients everywhere.

### ICT system interoperability challenges

One of the greatest barriers to the implementation of ICP is the lack of interoperability between the various ICT systems (16). The ICPs require that information related to the patient be prepared to thread their way through and seamlessly followed by different care teams for timely and coordinated interventions (17). Most probably, in most primary care settings, this information flow is hindered by different ICT systems, leading to fragmented care and thereby missed opportunities for timely intervention.

Lalani et al. (2) notes that integrated information and communication technology systems are vital to ensure that ICPs realise their full potential in enhancing care coordination. Without appropriate data sharing, the healthcare service providers get obliged to operate within silo systems, which tend to lower efficiency in care provision. Parry et al. (5) observed that poor interoperability associated with the ICT system in Tower Hamlets was associated with higher rates of active elective inpatient admissions. Therefore, it suggests poor care coordination despite the application of ICPs.

Interoperability of ICT systems necessitates investments into interoperable electronic health records (EHR) systems that will enable both sharing and exchange of patient information in a secure way with all care providers involved in an ICP (18, 19). In addition, integration of decision-support functionality within such systems can maximise the impact of ICPs by enabling real-time data-driven decisions for clinicians (20, 21). Among recent innovative technologies, blockchain solutions show promise in addressing current limitations of ICT interoperability for secure data sharing (19).

### A new concept: ICPs and predictive analytics for proactive care

While much of the discussion around ICPs has focused on standardisation and coordination, an area that remains largely unexplored is the integration of predictive analytics into ICP frameworks. This approach could shift the paradigm of ICPs from a reactive care-only approach—where interventions in care are based solely upon symptoms or conditions as they present themselves to a patient—to a proactive care model: guided, decision-based interventions based upon predictive models created directly from patient data.

Predictive analytics can analyse large volumes of health data to identify patterns and trends indicating future health risks. Thus, integrating such tools into ICPs might enable healthcare providers to predict possible complications well in advance and to intervene much earlier. For example, wearable devices could monitor a patient with chronic obstructive pulmonary disease (COPD), while predictive algorithms automatically alert the care team about subtle changes in respiratory function that presage an impending exacerbation (16).

Embedding predictive analytics in an ICP thus marks a new step towards personalised, data-driven healthcare. Such a novelty, disclosed herein, corresponds with the goals of ICP: improving patient outcomes, reducing admissions, and optimising resource utilisation. Accommodating other criticisms levelled by practitioners for the rigidity of ICPs, predictive analytics will also enable dynamic and personalised care plans that adapt in near real time to changing data inputs (22, 23).

Incorporating predictive analytics into ICPs might also offer a solution for some of the challenges related to the interoperability of the ICT systems. Advanced algorithms could run within existing EHR systems, providing real-time alerts and decision support without requiring far-reaching changes to the underlying infrastructure, thus making predictive tools as a cost-effective and scalable solution for improving the quality of ICPs in primary care (24, 25).

### Novel concept: patient co-design of ICPs

While much attention has focused on involving healthcare professionals in the design of ICPs, there is a growing awareness of the value of engaging patients as co-designers of care pathways. This perspective involves direct patient participation to be an active partner in shaping the ICPs in a novel approach for improving satisfaction and care outcomes for the patients.

Patient co-design goes beyond simply providing feedback; the patients get actively involved in decision-making about their care pathways. A model like this might increase treatment adherence and improve patient outcomes, as the care pathways are tailored to the needs, preferences, and values of the individual (26). Empowering patients to contribute to developing their care pathways fosters greater engagement in the care process, which, in turn, improves their health outcomes (27).

Recent studies have shown the prowess of patient co-design in chronic disease management, as patients are usually aware of the daily usefulness or limitations of current therapeutic modalities in managing their ailments (28). Thus, incorporating the patient's perspective into ICPs has the potential to give healthcare pathways that are more flexible and responsive to the unique challenges faced by a patient. This precept aligns with personalised medicine policies, where treatments are tailored to meet the needs of each patient (29).

It also serves to help reduce some of the resistance from health professionals in addition to improving patient-centred outcomes. Involving patients as co-designers in pathways allows providers to make them more flexible and patient-centred, reducing concerns about the rigidity of ICPs (30). This professionalism fosters an integrated relationship between patients and providers for improvements in the quality of care in general (31).

### Balancing the potential gains with challenges

While ICPs hold immense promise for improving healthcare, their practical implementation must balance the overall potential benefits and the professional resistive capabilities and ICT system limitations. An important consideration is whether the overall benefits of ICP—improved coordination of care and reduced medical errors that are associated with better patient outcomes—outweigh the burdens they impose on healthcare professionals and the healthcare system (32, 33). The adoption of ICPs usually requires operational and cultural changes within a practice. Such changes are met with resistance more often than not, exclusively when the perceived benefits of the ICPs are abstract or long-term, while the challenges brought forth, in such as administrative burden and workflow changes, are real and felt immediately. All these changes can feel overwhelming for every healthcare provider, especially in primary care, and often result in resistance to adopting the ICPs, no matter how much they could help in improving patient care.

Moreover, the infrastructural requirements for implementing ICPs, specifically ICT system interoperability, may offer formidable barriers. Many health systems rely on antiquated or incompatible digital platforms, preventing the easy flow of patient data across multidisciplinary teams. Without proper ICT infrastructure, the efficiency gains that ICPs promise, such as real-time data sharing and integrated decision-making, become unattainable. This indicates that special attention has to be given not only to the design issues of the ICPs as such but also to the preparedness of technological systems on which these ICPs rely. While analysing the balance between potential benefits and operational challenges of ICP implementation, the costs related to upgrading ICT systems, training health professionals, and maintaining these systems must be accounted for.

Carlile's work on overcoming knowledge boundaries sheds light on this balancing act. To quote Carlile, “building collaboration in complex systems such as ICP is possible” (6). His study thus infers that successful implementation requires bridging gaps among professional groups and fostering shared responsibility among all the involved stakeholders—from clinicians to ICT specialists—to establish a sense of collective investment across the board (34). Such a culture can help reduce some of the barriers to ICPs, enabling them to move from perceived threats to professional autonomy into collaborative tools that enhance the quality of care. This cultural shift becomes necessary not only for overcoming any professional resistance but also for developing and using the ICT systems in a way that enables the successful integration of ICPs into everyday practice.

## Discussion

While ICPs hold great promise for significant enhancement in healthcare provision, especially at the primary care level, attaining these require overcoming many systemic stumbling blocks. Each stakeholder, including clinicians in both primary and secondary care, patients, integrated care boards (ICBs), and the National Health Service (NHS) itself, has a different orientation. The concerns of each stakeholder group have to be addressed to create an environment where ICPs can be at their best in providing coordinated, efficient, and patient-centred care (Table 2).

**Table 2:** Addressing key challenges in ICP implementation: a comprehensive table of solutions and innovations.

Challenge/impediment with supporting references	Proposed solution	Creative and analytical insights	Futuristic or unconventional innovations
Professional resistance to ICPs Perceived loss of clinical autonomy due to rigid protocols, limiting individual patient-centred decisions (9–14)	Involve clinicians in ICP co-design to ensure flexibility and emphasise benefits like reduced errors and improved care	Rethinking ICPs as decision-support tools rather than prescriptive mandates can shift the mindset of healthcare professionals. This change allows ICPs to become dynamic frameworks that enhance autonomy by reducing decision-making fatigue	“Neuro-ergonomics for Clinician Support” Incorporate neuro-ergonomics to measure cognitive load in real-time and adjust ICP decision-support to match the mental strain of clinicians, optimising their workflow while preserving autonomy
ICT system interoperability Fragmented ICT systems hinder data sharing, disrupting coordinated care and delaying interventions (6, 8, 16–19, 21)	Invest in interoperable EHR systems and consider blockchain for secure data sharing	Viewing ICPs as “ecosystems” where seamless data flow across platforms becomes the lifeblood of care coordination. Blockchain can be revolutionary here, providing a transparent, tamper-proof data exchange that enhances real-time decision-making	“Quantum Computing for Interoperability” Leverage quantum computing to process large, complex datasets from disparate systems instantaneously, creating universal compatibility between different ICT systems and revolutionising real-time data sharing
Standardisation vs. personalised care ICPs may prioritise standardisation over individualised patient care, limiting flexibility in complex cases (3, 4, 12, 20, 28)	Develop adaptive ICPs with customisable care plans to accommodate patient preferences and complex cases	Reframing standardisation as the “scaffold” that supports personalised care. ICPs should be seen as flexible frameworks that adapt dynamically to individual patient data, evolving from static guides into responsive systems that learn from each case	“AI-Driven Hyper-Personalisation” AI-driven hyper-personalisation that continually adapts ICP protocols based on a patient's real-time genetic, behavioural, and environmental data to provide care as uniquely tailored as a fingerprint
Administrative burden Additional documentation and compliance requirements increase the workload for clinicians (11, 12, 15, 17)	Automate documentation and integrate ICP tools into EHR systems to streamline processes and reduce manual tasks	Turn administrative tasks from burdens into assets by integrating artificial intelligence and automation, which can assist clinicians in real time. Clinicians can focus on patient care while still meeting compliance requirements seamlessly	“Augmented Reality for Documentation” Use augmented reality (AR) glasses that automatically record patient interactions, annotate key medical data, and integrate seamlessly into EHR systems, freeing clinicians from manual documentation tasks
Cost and resource allocation Upfront costs for implementing ICPs, including technology upgrades and training, may deter adoption (7, 19, 34)	Perform cost–benefit analyses to demonstrate long-term savings from reduced readmissions and better resource management	Think of ICP adoption as an “investment in precision.” Aligning resource allocation with patient needs based on predictive analytics transforms ICPs into precision tools, targeting interventions where they are most likely to make an impact	“Tokenised Healthcare Incentives” Create a blockchain-based token economy where healthcare providers are rewarded with tokens for successfully using ICPs to reduce readmissions and optimise resource use, which can be redeemed for training or equipment upgrades
Predictive analytics integration Lack of use of predictive analytics in ICPs to anticipate complications and provide proactive care (16, 22–25)	Integrate machine learning and wearable devices to monitor real-time data, enabling early interventions	Predictive analytics represent a paradigm shift. Instead of ICPs reacting to patient symptoms, they should evolve into anticipatory systems that enable pre-emptive care. ICPs should evolve into responsive systems that adapt based on real-time data	“Digital Twin Technology for Health” Create digital twins of patients, using real-time data from wearables and health records to simulate and predict future health scenarios, allowing proactive adjustments to ICPs and earlier interventions for critical conditions
Patient involvement in ICP co-design Lack of patient input in ICP design reduces adherence and satisfaction (26–30)	Involve patients directly in co-design sessions for more personalised pathways, supported by digital engagement tools	Engage patients as “co-creators” of their healthcare journeys. When patients contribute their lived experiences to ICP design, they are not just passive recipients of care—they become active partners, improving adherence and satisfaction	“Biometric Feedback in ICPs” Use biometric feedback (heart rate, blood pressure, etc.) integrated into patient portals that allow patients to see the real-time effects of their health decisions, increasing ownership and engagement with their ICP
Cultural shift for ICP adoption Healthcare professionals resist collaboration and cultural change required for ICP adoption (6, 34)	Promote interdisciplinary collaboration, educate on the long-term benefits of ICPs, and provide incentives for teamwork	Reposition ICPs as catalysts for building a “collaborative culture” within healthcare. Fostering a sense of shared responsibility ensures that every stakeholder sees value in the transformation, creating a more united healthcare delivery model	“Gamified ICP Adoption” Develop a gamified platform where healthcare teams are awarded points for successful ICP adoption, with leader boards and collaborative goals that encourage friendly competition and cultural change towards interdisciplinary collaboration

For primary care clinicians, who commonly serve as the point of entry for patients and are often responsible for a wide range of health conditions, this rigidity of ICPs may threaten their ability to provide appropriate management for their individual patient needs. Resistance among professionals is usually viewed as stemming from standardisation, which is perceived to reduce the clinician's areas of autonomy (9).

These issues can be addressed only by designing ICPs to allow a certain degree of flexibility in their adoption so that primary care clinicians can deviate from the protocol where necessary. The challenge lies in striking a balance between providing structured, evidence-based care and supporting individualised decision-making, which is at the heart of primary care. By engaging clinicians as co-designers of ICPs, they thus can contribute to pathways that are standard yet stable, empowering them rather than making them feel constrained by the system (13). Such engagement can also allow them to view ICPs not as backward protocols but, rather, as frameworks designed to facilitate quality care by reducing errors and ensuring consistency.

For secondary care clinicians, especially those in hospitals or specialist settings, ICPs provide an opportunity to improve coordination with primary care. One of the major challenges in healthcare generally is the fragmentation between different levels of service, which can result in duplicated tests, delayed diagnoses, and suboptimal patient outcomes (18).

ICPs serve as the bridge that would ensure a smooth transition of care from the primary to the secondary level of care and enable all providers to work within the same standardised care plan. However, the problem of effective communication between the two sectors still remains. Secondary care must receive real-time information about progress and treatment from primary care (17). This implies that the ICT systems used between the two sectors should allow for interoperability between them to ensure seamless information exchange. That is, secondary care clinicians are involved in their development to taken into consideration the complexities of specialty care.

While ICPs are for the benefit of patients, their voices are often not raised in shaping the care pathways. Engaging patients in the co-design of ICPs may improve understanding and satisfaction with the care delivered (26). Evidence shows that patients who participate in their plan of care are more likely to adhere to treatments, resulting in improved health outcomes.

From a patient's viewpoint, the core challenge lies in receiving services in diverse settings from multiple providers, whatever the nature of the conditions—chronic or complications arising from multimorbidity. ICPs informed by their input through co-design can thereby be tailored to meet individual preferences and needs, with the active participation of patients in their care. This approach will help demystify the healthcare process and make it much easier for the patients to understand their current and future care plans; importantly, it also encourages patients to take responsibility for their health (28). Some of the digital engagement tools implemented through ICPs include patient portals, which were designed to provide real-time updates and enhance transparency in communication between patients and providers (31).

ICBs are critically involved in the implantation and monitoring of ICPs. Their major role is to ascertain that the various levels of care, such as primary, secondary, and community, are aligned towards common goals. There is interference by ICBs in coordination across various healthcare providers and in ensuring that each stakeholder adheres to the care pathways.

In this regard, ICBs must invest in robust ICT systems that provide necessary communication and data sharing across care settings. This ensures coordinated care delivery without delays or duplications. In addition, ICBs need to ensure that performance metrics elicit monitoring and evaluation of ICPs so that bottlenecks in the system may be identified with your subsequent corrective actions on them (19). Moreover, it will involve clinicians and patients in developing and refining these pathways to ensure that ICPs are theoretically sound and practically effective.

As a national healthcare system, the NHS bears the added responsibility of ensuring that ICPs are scalable and sustainable across regions and health settings. One of the major challenges facing the NHS concern is the need for heavy investment in ICT infrastructure. Unless interoperable systems that share patient data across all care settings are involved, the full potential of ICPs will hardly be achieved (21).

The NHS also has to deliver a cultural change in the way healthcare is provided. To date, care is provided by silos of primary, secondary, and community care providers that often work in isolation from one another. For ICPs to succeed, the NHS needs to develop a more integrated approach where care is regarded as a continuum, with all providers adhering to the same overall care plan. Policies and incentives that promote collaboration and teamwork across care settings will be critical in achieving this shift (34).

Finally, the NHS should be concerned about the long-term ICP sustainability. While the initial investments in both technology and training are huge, the longer-term benefits that will be derived from better patient experiences, complemented by reduced hospital admission rates and more cohesive coordination of care, would thus create a balance with those costs. Regular re-evaluation and adjustment of real-world, evidence-based ICPs will guarantee payoff in relevance and effectiveness in a constantly changing healthcare environment because of changing parameters in the complex environment (32).

## Conclusion

Seamless implementation of ICPs requires unified efforts to curtail concerns and difficulties encountered by all concerned stakeholders. Primary clinicians should be empowered to use ICPs as flexible tools that accommodate personalised care. Secondary care providers should be integrated into the continuum of care through interoperable ICT systems that allow timely communication. Patient engagement in the co-design of care pathways is necessary to make them active partners in their healthcare journey. ICBs must guarantee coordination and accountability across healthcare settings, and the NHS should lead the way to promote a culture of collaboration and sustainability.

Because they address many of these challenges in many directions, ICPs can be truly transformative tools for improvement in the delivery of healthcare. An ICP should be able to provide patients with coordinated, high-quality, efficient, patient-centred care through innovative engagement and integration within the system.
